# Ketone body, as an emerging modulator of metabolic reprogramming and epigenetics in breast cancer 

**DOI:** 10.22038/ijbms.2025.84064.18185

**Published:** 2025

**Authors:** Fatemeh Mehdikhani, Sadegh Rajabi, Maryam Mohammadlou, Marc Maresca, Rasoul Baharlou

**Affiliations:** 1 Department of Clinical Biochemistry, School of Medicine, Shahid Beheshti University of Medical Sciences, Tehran, Iran; 2 Traditional Medicine and Materia Medica Research Center, Shahid Beheshti University of Medical Sciences, Tehran, Iran; 3 Cancer Research Center, Semnan University of Medical Sciences, Semnan, Iran; 4 Aix Marseille University, CNRS, Centrale Marseille, iSm2, 13013 Marseille, France; 5 Department of Immunology, School of Medicine, Semnan University of Medical Sciences, Semnan, Iran

**Keywords:** Breast cancer, Epigenetics, Histone modifications, Ketone bodies, Metabolism

## Abstract

The metabolic profile of cancer cells, notably their reliance on glucose as a primary energy source for proliferation, sets them apart from normal cells. This metabolic dependency may significantly affect their invasive potential when an adequate glucose supply is available. Moreover, emerging evidence underscores the critical role of metabolism in determining the epigenetic landscape of cells. To limit the glucose supply and alter cancer cell metabolism, researchers have investigated ketogenic diets as an alternative energy source for cancer cells and providing a promising strategy to combat cancers. However, controversial findings in the literature suggest a direct relationship between the use of ketone bodies in cancer cells and the augmentation of invasiveness. Additionally, studies indicate that using ketone bodies as an energy source can influence the epigenetic patterns of tumor cells. Breast cancer cells show a unique metabolism by which the cancer cells adapt to various conditions. This paper aims to review the metabolic characteristics of breast tumors, focusing on the ketone body metabolism in this cancer and the complex interplay between ketone bodies and the epigenetic changes in this cancer.

## Introduction

**Figure 1:**
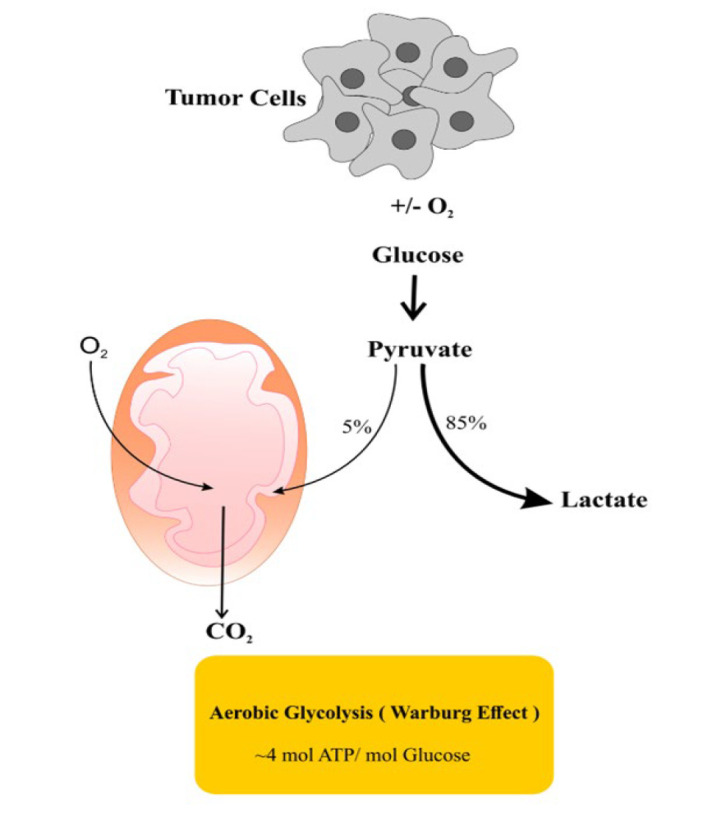
Schematic representation of the Warburg effect in cancer cells

Cancer is the second leading cause of death worldwide, after cardiovascular diseases (1). Breast cancer has been reported as the most common type of cancer in the world, and it is expected that over 3 million new cases will be affected by 2040 (2). Based on cellular markers, breast cancer can be categorized into three main types: those with estrogen (ER) or progesterone receptors (PR), those with overexpression of epidermal growth factor-2 (HER-2), and those lacking ER, PR, and HER-2, known as triple-negative breast cancer (TNBC) (3-5). These markers also determine treatment strategies. Based on the immunohistochemistry markers and microarray studies, breast cancer can be further classified into five subgroups: Luminal A, characterized by positive ER or PR and negative HER-2; Luminal B, with positive ER and HER-2 or have high levels of Ki-67; HER-2 overexpressing subtype, displaying negative ER and PR but positive HER-2; Basal-like subtype, with negative ER, PR, and HER-2 but positive cytokeratin 6.5; and Normal breast-like subtype (6-8). One of the specific features of cancer cells is their metabolism, which is significantly different from that of normal cells. Cancer cells obtain the ability to employ the Warburg effect in both oxygen-rich and oxygen-poor environments, consuming a large amount of glucose to provide energy (Figure 1) (9). Several studies have investigated the metabolic profiles of breast tumors by focusing on their different aspects such as glutamine uptake, lipid metabolism, and glycolytic hyperactivation. These investigations have revealed distinct metabolic alterations within breast tumors (10-13). Different subtypes of breast cancer exhibit distinct metabolic adaptations. For instance, TNBC significantly depends on glycolysis, even in the presence of oxygen. This reliance on glycolytic activity contributes to TNBC’s aggressiveness, rapid proliferation, and resistance to treatment compared to other subtypes (14). Similarly, basal-like breast tumors also show a high dependence on glycolysis, whereas luminal breast cancers tend to rely more on oxidative phosphorylation for their energy needs (15). According to the new evidence supporting altered metabolism’s effect on tumor cell fate, researchers are interested in investigating cancer metabolism to find possible ways to suppress cancer cell growth (16). However, cancer cells are highly adaptive to metabolic changes, making them challenging targets for cancer therapy. Consequently, disrupting metabolic pathways associated with the Warburg effect by inducing nutrient-deprived conditions has encountered significant challenges and limitations (17, 18). While cancer cells generally depend on glucose, some tumor cells with mitochondrial dysfunction cannot metabolize ketone bodies (19). Thus, recent research has proposed that reducing glucose intake and replacing it with a ketogenic diet may be a viable therapeutic strategy to inhibit tumor progression (20). For example, a study using a VM-M3 mouse model of metastatic cancer demonstrated that ketogenic diets reduced tumor progression and increased survival rates (21). However, some contradictory evidence suggests that ketone bodies may positively influence cancer growth. For instance, Martínez-Outschoorn *et al*. revealed that stromal cells within the tumor microenvironment can supply ketone bodies for breast tumor growth (22). Besides, several lines of evidence have implied the presence of a critical connection between diet and epigenetic alterations (23). In earlier investigations, mutations in specific genes, notably BRCA1 and BRCA2, were identified as the primary instigators of breast cancer. However, new research has increasingly unraveled the role of epigenetic modifications in promoting the growth and progression of breast cancer (24). Recent evidence has also shown an intricate relationship between ketone bodies and epigenetics. For example, beta-hydroxybutyrate (BHB) has been discovered to play a role in post-translational modifications, particularly those that affect histones (25). Cancer cells generally show considerable and uncontrollable growth, necessitating a higher demand for energy than normal cells. Consequently, metabolic reprogramming emerges as a pivotal phenomenon in cancer cells, enabling them to fulfill their energy needs. Initially, cancer cells orchestrate glycolytic pathways to provide energy demands while inhibiting oxidative phosphorylation (26). Subsequently, they convert this metabolic paradigm to increased tricarboxylic acid (TCA) cycle activity to obtain enough energy to support their proliferation (27, 28). The primary purpose of the present review is to describe the metabolic characteristics of breast tumors, focusing on the ketone body metabolism in this cancer. Moreover, this review summarizes prominent epigenetic changes in breast cancer and the interplay between epigenetics and ketone body metabolism.

### Metabolic reprogramming in breast cancer

**Figure 2:**
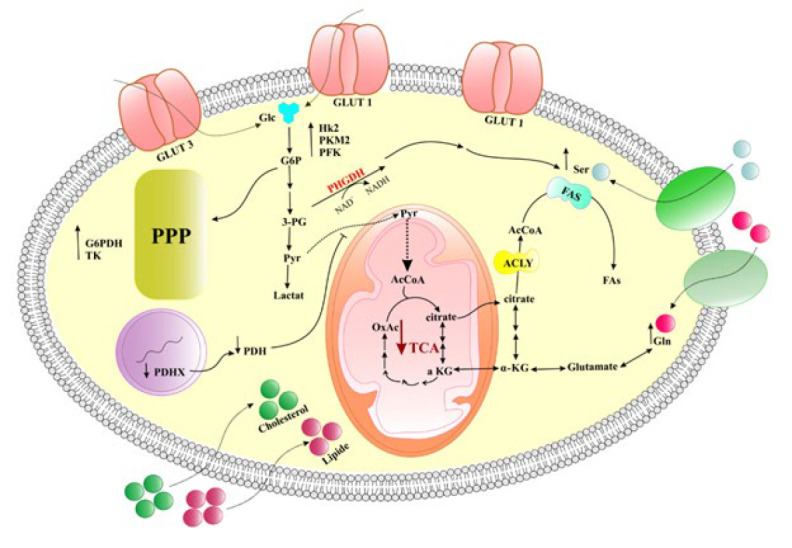
Overview of metabolic reprogramming in breast cancer cells

 Tumor cells can rearrange their metabolic pathways, orchestrating the processes that support their uncontrolled proliferation. This phenomenon is metabolic reprogramming, by which cancer cells save the energy resources essential for their survival and progression (29). Among a variety of alterations in cancer cell metabolism, the most important ones are the increased glycolytic activity, the up-regulation of amino acid and lipid metabolism pathways, increased glutaminolysis, induction of the pentose phosphate pathway, facilitation of macromolecular biosynthesis, and mitochondrial biogenesis (29-35). Through complex metabolic reprogramming, cancer cells effectively utilize various nutrient sources to handle unlimited proliferation. In breast cancer cells, such a dysregulated metabolism is characterized by a significant elevation in glycolytic activity (Figure 2) (36-39). Increased glycolysis supports the unrestrained proliferation of breast cancer cells (40). It has been evidenced that the major role player in glucose uptake is Glucose transporter 1 (GLUT), which is overexpressed in TNBC and is correlated with unfavorable prognosis (41, 42). Moreover, elevated expression levels of GLUT1 and GLUT3 have been observed in high-grade breast cancers (43). Other important role players in aggravating this metabolic phenotype are hyperactivated glycolytic enzymes. For example, hexokinase 2, a critical enzyme in the glycolytic pathway, is up-regulated in breast cancer to enhance glycolysis in these cancer cells (44, 45). Elevated activity of pyruvate kinase M2 and phosphofructokinase have been reported to be associated with diminished survival rates and increased risk of metastasis in breast cancer patients (46-49). 

 These cells have shown a significant decrease in the function of the TCA cycle. For example, breast tumors show a significant decrease in the expression of the PDHX component of pyruvate dehydrogenase (PDH). This enzyme is responsible for channeling metabolites from glycolysis into the TCA cycle. Reduced expression of PDHX has been reported to be correlated with adverse prognosis (50). 

A plethora of evidence indicates activation of the pentose phosphate pathway (PPP) in cancer cells. This metabolic pathway produces NADPH and ribonucleotide intermediates, which are crucial for lipid biosynthesis and glycolysis (51). PPP-related enzymes, including glucose 6-phosphate dehydrogenase (G6PD) and transketolase, have been revealed to be hyperactivated within breast cancer cells (52). In breast tumors with HER-2 overexpression, a significant increase in the expression levels of PPP enzymes has been described (53). 

Metabolism of some amino acids, such as glutamine and serine, is necessary to provide breast tumor growth. For example, glutamine is an essential amino acid for the metabolic demands of breast tumors (54). TNBC has been reported to have elevated levels of glutaminase, an enzyme that catalyzes the conversion of glutamine to glutamic acid (55). This augmented expression of the enzyme may suggest the TNBC cells’ dependence on exogenous sources of glutamine for survival (56). However, luminal tumors have been shown to up-regulate glutamine synthetase, an enzyme involved in glutamine synthesis (57). Serine is another amino acid that can be synthesized in breast cancer cells via the activity of the 3-phospho-glycerate dehydrogenase (PHGDH) enzyme, which is up-regulated in breast cancer (58, 59). The elevated expression of PHGDH and subsequent availability of serine are associated with the amplified growth in breast tumors (60). 

Tumor cells also need lipids for the synthesis of the cancer cell membranes. To meet this goal, elevated lipid synthesis and the uptake of external cholesterol and lipids are necessary (61, 62). Fatty acids essential for the formation of the cell membrane in breast cancer cells are synthesized by the up-regulation of fatty acid synthase (FAS) (63). FAS expression is markedly elevated in breast tumors and is associated with poor prognosis and tumor relapse (64). The expression patterns of FAS vary between breast cancer subtypes, with a downregulated level in TNBC and an up-regulated expression in HER2^+^ tumors (65). Additionally, breast cancer cells display a high propensity for the uptake of lipids and some molecules, such as choline, to provide building blocks for the formation of cell membranes. Both TNBC and luminal tumors exhibit incremented choline uptake, which is metabolized into phosphocholine and phosphatidylcholine (66, 67). Then, phosphatidylcholine is converted into choline and phosphatidic acid by phospholipase D (PLD). Phosphatidic acid has been unraveled to be associated with invasiveness and metastasis in breast tumors (68). Additionally, breast tumors with elevated proliferative activity often exhibit increased expression levels of PLD, highlighting the significant role of lipid metabolism in driving breast cancer progression (68, 69).

### Some prominent epigenetic modifications in breast cancer

**Figure 3:**
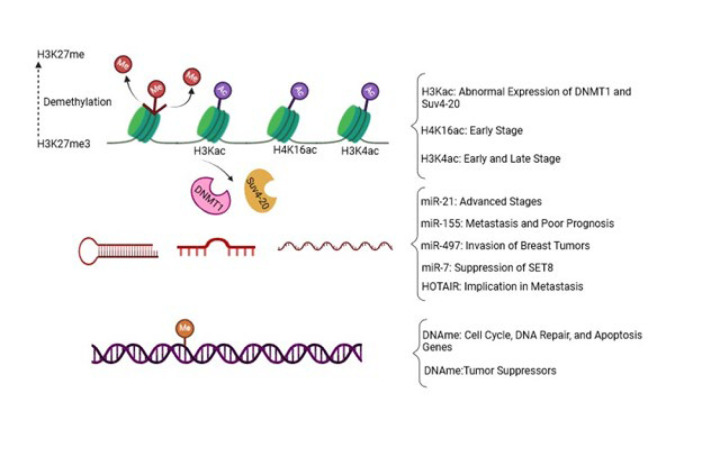
Some epigenetic modulators in breast cancer. This figure illustrates several key epigenetic alterations observed in breast cancer cells

Recent evidence has shown that the primary promoting factor of breast cancer development lies in epigenetic alterations (70). It has been established that the intricate interplay between mutations in oncogenes, tumor suppressor genes, and epigenetic changes may lead to the enhanced metastatic potential of breast tumors (71, 72). Epigenetic alterations orchestrate specific gene expression profiles, promoting uncontrolled growth and metastatic propagation of breast cancer (24). Describing all of the epigenetic alterations of breast cancer here is exhaustive. Therefore, we point out some prominent epigenetic changes in breast tumors in this section (Figure 3). 

Recent investigations revealed that different breast cancer cell phenotypes show unique histone modification patterns. For instance, a subpopulation of drug-sensitive breast cancer cells bears a demethylated form of H3K27me3 (73). The activity of histone acetyltransferases (HATs) leads to the up-regulation of the Catechol-O-Methyltransferase gene, which is a well-characterized risk factor for breast cancer. Augmented activity of HATs also impedes the proliferation of estrogen-dependent breast cancer cells (24). Two histone modifications, including acetylation and methylation, have been numerously reported in breast cancer (74-76). In breast cancer cells with HER-2 overexpression, an increased level of lysine acetylation in H3 and H4 has been underscored (76, 77). An *in vitro* study indicated that the elevated acetylation of H4 was associated with abnormal expression of DNA methyl transferase 1 (DNMT1) and histone methyl transferase Suv4-20, two key epigenetic modifiers (78). In the early stages of breast cancer, the level of H4k16ac is significantly decreased (79). The H3K4ac mark is associated with both early and late stages of breast cancer (80). Dysregulated histone acetylation can modulate the expression of both oncogenes and tumor suppressor genes. Additionally, it plays a role in the expression of other genes related to cell cycle, apoptosis, and cell growth in breast cancer (81). Inhibiting histone deacetylases (HDACs) has been shown to suppress breast cancer, making it a potential strategy for the treatment of breast cancer (80). Several investigations have suggested that the incidence of breast cancer is linked to abnormalities in histone modifications and transcription factors (81). A study conducted on MCF-7 cells by Jin and colleagues revealed the association of three histone modifications—H3K27me2, H3K27ac, and H3K4me1—with changes in the expression of genes associated with breast cancer. Specifically, alterations in H3K27me2 were found to play a significant role in the initiation and progression of breast carcinoma (81). Moreover, k9me3/k14Ac or k9me3 accumulation was detected in breast tumor cell lines, while the k14Ac pattern was absent in primary cell lines (82). Furthermore, during the early stages of breast tumor development, a marked increase was shown in the histone modifier LSD1, which removes methyl groups from H3K9 and H3K4 (83). DNA methylation is another epigenetic modification in CpG islands of gene promoters and leads to gene silencing (84). Breast tumors often exhibit high levels of DNA hypermethylation, resulting in the silencing of critical genes of the cell cycle (e.g., p16, RASSF1A), DNA repair (e.g., BRCA1), and apoptosis (e.g., HOXA5, TMS1) (85-87). Furthermore, methylation of tumor suppressor genes such as CDH1, APC, CTNNB1, and 14-3-3 Sigma is associated with breast cancer growth (88, 89). A study on 179 breast cancer samples revealed hypermethylation of two promoter regions (Alu and LINE-1), which were correlated with the HER2^+^ breast cancer subtype (90). Additionally, several studies have provided evidence that DNA hypermethylation occurs in the early stages of breast tumor development (91). Moreover, Studies have shown that hypermethylation of promoters in tumor suppressor genes, such as CCDN2 (Cyclin D2), P16 (cyclin-dependent kinase inhibitor 2A), ATM (ataxia telangiectasia mutated), RASSF1A (Ras Association Domain Family Member 1A), APC (Adenomatous Polyposis Coli), and BRCA1 (breast cancer gene 1), is commonly observed in breast cancers. This hypermethylation contributes to breast tumor progression by inhibiting apoptosis and disrupting cell cycle regulation (91-93). In contrast to DNA hypermethylation, which occurs at specific gene promoters, widespread hypomethylation throughout the genome results in genomic instability and triggers the activation of oncogenes in breast tumors (93).

The third epigenetic role-player is miRNA, which is generally dysregulated in breast tumors (94). For instance, miR-21 is significantly up-regulated in advanced stages of breast cancer, while increased expression of miR-155 is associated with metastatic behavior and poor prognosis (95, 96). Moreover, miRNA-497 has been shown to affect cell proliferation and invasion in breast cancer by targeting cyclin E1 (97). MiR-7 has been identified as an important role-player in suppressing the activity of a histone methyltransferase known as SET domain containing 8 (SET8) within invasive breast cancer cells. SET8 is primarily responsible for catalyzing methylation of the histone H4 at lysine 20 (H4K20me). Consequently, down-regulation of H4K20me by miR-7 impedes the epithelial-mesenchymal transition (EMT) (98). 

Other epigenetic modulators implicated in breast cancer progression are long non-coding RNAs (lncRNAs), characterized by their length exceeding 200 nucleotides. For example, HOTAIR is an lncRNA involved in the transforming growth factor beta (TGFβ)- induced metastasis in breast tumors (99).

### Ketogenic diet composition

A ketogenic diet is characterized by increased fat consumption, reduced carbohydrate intake, and moderate protein intake (100). Different cells primarily rely on glucose as their primary energy source. When glucose availability is limited, body cells switch to ketogenesis to meet their energy needs (101). Three forms of ketone bodies in the body are beta-hydroxybutyrate (BHB), acetone, and acetoacetate (102). There are various ketogenic diets, including the classic ketogenic diet, the modified Atkins diet, the very low-energy ketogenic diet, and the ketogenic Mediterranean diet (103). The classic ketogenic diet, which was initially developed for epilepsy patients, is characterized by a strict macronutrient composition of protein (6%), carbohydrates (4%), and fat (90%) (104). The modified Atkins diet consists of protein (30%), carbohydrates (5%), and fat (65%) (105, 106). The very low-energy ketogenic diet significantly restricts carbohydrate intake, mimicking the effects of fasting. It typically comprises 43% protein and 44% fat (107). The Ketogenic Mediterranean diet emphasizes a very low carbohydrate intake and includes fish, lean meats, walnuts, salads, and olive oil (108-112). In some conditions, Mediterranean diets recommend herbal extracts (113).

### The role of ketone bodies in breast cancer metabolism and therapy

Due to the dependence of breast cancer cells on glucose as a primary energy source, the role of ketone bodies in the metabolism of breast cancer remains unclear (114).

***In vitro studies***

An *in vitro* study on MDA-MB 231 and MCF-7 cells revealed that breast cancer cells could not utilize ketone bodies as an energy source for growth (114). However, Bonuccelli *et al*. researched MDA-MB231 cells, revealing that BHB contributed to increased tumor growth (115). Another study, focusing on MCF-7 and immortal fibroblast hTERT, demonstrated an up-regulation of the enzymes involved in the ketone body production and consumption within the stroma of breast cancer cells. Additionally, it was revealed that catabolic fibroblasts in the tumor microenvironment trigger tumor growth by producing and secreting ketone bodies (116). This observation suggests that ketone bodies play a critical role in tumor progression and metastasis. Besides, another study on the MDA-MB 231 cell line provided evidence to highlight that ketone bodies can potentially stimulate both the growth and metastasis of breast cancer (117). A comprehensive study involving various breast cancer cell lines, including BT20, BT474, HBL100, MCF-7, MDA-MB 231, MDA-MB 468, and T47D, revealed that treatment of the cell lines with BHB had remarkable effects on cell proliferation, chemotherapy response, or radiation sensitivity *in vitro*. Furthermore, BHB treatment-induced changes in BT20 breast cancer cells’ energetic phenotype have no significant effect on other cell lines (118). Maldonado *et al*. investigated MCF-7 and T47D cell lines suffering from glucose deprivation in the presence of BHB. Their data revealed a considerable decrease in the proliferation rate of these breast cancer lines. This observation suggests that these cell lines may not effectively utilize BHB as an energy source when glucose is limited (119). Additionally, a study demonstrated that the viability of MCF-7 cells decreased when glucose was absent, and ketone bodies such as acetoacetate and BHB were available. The above data may reveal that cancer cells do not predominantly rely on ketone bodies as energy sources (120).

***In vivo studies***

An *in vitro* and *in vivo* study demonstrated an induced expression of monocarboxylate transporter 2 (MCT2), responsible for ketone body uptake, in breast tumors. This expression was associated with increased invasiveness of tumor cells (121). Research suggests that a ketogenic diet exhibits anti-cancer growth properties and exerts a tumor-suppressive effect on breast cancer (122). Besides, in mouse models of breast cancer, a ketogenic diet has been shown to enhance the efficacy of targeted therapies such as PI3K inhibitors, potentially overcoming drug resistance (123). Furthermore, Dai *et al*. proposed that the exposure of breast cancer cells to a ketogenic diet in a mouse model could induce the activation of AMP-activated protein kinase (AMPK), which may improve the efficacy of anti-CTLA-4 immunotherapy by reducing Programmed Cell Death Ligand 1 (PD-L1) expression and enhancing the expression of antigen-presenting genes and type-1 interferon (124). Salem and colleagues showed that the knockdown of BRCA1 in tumor-associated stromal fibroblasts (shBRCA1 fibroblasts) leads to a significant increase in ketone body production. Co-culturing and co-injected BRCA1-deficient fibroblasts with MDA-MB-231 cells in a mouse model enhanced mitochondrial activity in cancer cells to support their proliferation (125).

***Clinical trials***

Several investigations have been conducted to assess the effects of ketogenic diets on breast cancer patients. A ketogenic diet improved metabolic parameters, body composition, and overall survival in breast cancer patients, according to a recent study (126). Klement *et al*. performed a clinical study involving breast cancer patients who are under radiotherapy. The findings indicated that ketogenic diets led to notable improvements in body composition, metabolic profiles, and participant-reported quality of life (127). Among patients with locally advanced and metastatic breast cancers, it was observed that ketogenic diets did not affect the quality of life, physical activity, dietary intake, or biomarkers (thyroid hormones, electrolytes, albumin, LDH, or ammonia) over 12 weeks. However, after 6 weeks, the group adhering to the ketogenic diet showed higher global quality of life and physical activity levels than the control group (128). Another clinical trial aimed to evaluate the feasibility and metabolic impacts of a personalized, well-formulated ketogenic diet (WFKD) among women diagnosed with stage IV metastatic breast cancer (MBC) who are under chemotherapy. The findings indicated that women with MBC undergoing chemotherapy can safely achieve and maintain a state of ketosis through a ketogenic diet while improving metabolic health outcomes over six months (129). Evidence suggests that combination of ketogenic diets with chemotherapy regimens may result in positive outcomes for patients with Triple-negative breast cancer. In this case study, the patient tolerated the treatment well and experienced significant improvement in quality of life (130). Although ketone bodies exert contradictory effects on cancer cells, researchers are focusing on this area of research to elucidate the net effect of ketone bodies on these cells. In a study conducted by Khodabakhshi et al., treatment of breast cancer patients with ketogenic diets for 12 weeks resulted in a significant decrease in tumor size and stage (131). The effect of ketone bodies on breast cancer appears to depend on the specific subtypes of this cancer and other potential contributing factors. This can determine the way to find efficient strategies for the treatment of breast cancer using ketogenic diets. Table 1 summarizes the studies that deal with the effect of ketone bodies on different models of breast cancer. 

### The interplay between ketone bodies and epigenetic modifications in breast cancer

Based on the literature, alterations in metabolism and epigenetic modifications are two prominent features of cancers (132-134). Metabolites produced in metabolic pathways can serve as substrates or cofactors for epigenetic alterations. Besides, those genes related to metabolism and enzymes involved in the metabolic pathways of cancer cells may exhibit differential expression due to changes in the epigenetic factors. Consequently, these two processes have a reciprocal interaction, which may make tumor cells more invasive and aggressive (135, 136).

**Figure 4:**
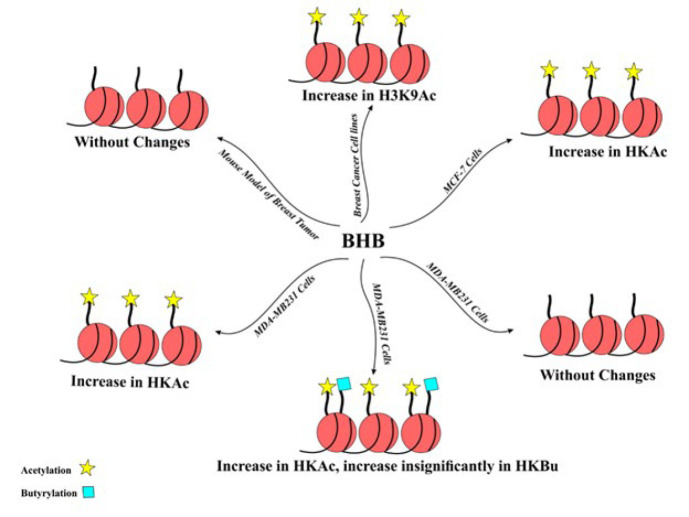
Effect of BHB on histone modifications in breast tumor cells

The role of ketone bodies in epigenetic regulation and gene expression introduces a further level of intricacy to our knowledge, offering significant perspectives on the effect of ketone bodies on cancer cells (137). Exploring their role in regulating epigenetic processes and gene expression provides valuable insights into the multifaceted effects of ketone bodies on cancer. A study demonstrated that combining a ketogenic diet with immune checkpoint blockade (ICB) therapies in prostate cancer led to the inhibition of histone deacetylases (HDACs) by BHB. This inhibition resulted in epigenetic reprogramming and increased MHC class I molecules expression on the tumor cell surface, making them more recognizable to CD8+ T cells. As a result, the immune system more effectively targets and destroys the tumor cells (138). Research indicates that HDAC3 is overexpressed in breast cancer, leading to the silencing of tumor suppressor genes through its histone deacetylase activity. This process promotes tumor survival and progression (139). Since BHB has an HDAC inhibitory activity, it may potentially counteract this effect by suppressing HDAC3, helping to restore the expression of tumor suppressor genes and hinder cancer progression. However, this hypothesis requires direct experimental evidence. An *in vitro* and *in vivo *study found that drug-resistant patient samples exhibited elevated levels of H3K79 methylation and H3K27 acetylation, which was significantly inhibited by BHB, leading to a reversal of Oxa resistance in both *in vitro *and *in vivo* models of CRC (140). In human breast cancer cells, using BHB has resulted in the elevation of H3K9ac and the up-regulation of tumor-promoting genes such as IL-1beta and LCN2, contributing to increased tumorigenesis (141). In another investigation, treatment of MCF-7 cells with BHB induced alterations in gene transcription compared to untreated cells. Additionally, there was a significant increase in histone acetylation. The findings of that study provided evidence to highlight the potential of ketone bodies to change the transcriptional gene profile in MCF-7 cells (117). In a study utilizing a mouse model of breast cancer, the administration of BHB was found to stimulate tumor growth. However, there was no significant effect on histone acetylation levels (142). Goudarzi et al. demonstrated that treating the MDA-MB-231 cells with BHB increased histone acetylation. Furthermore, their study revealed a minimal elevation in histone butyrylation (143). Another study revealed that long-term treatment of MDA-MB231 cells with BHB did not increase histone acetylation and butyrylation (144) (Figure 4). These paradoxical findings may arise from the heterogeneity of breast cancer cells or the ability of cancer cells to adapt and evade genetic or epigenetic changes. 

Cancer cells consume glucose and produce lactate to acquire their essential energy either in the presence of oxygen or in its absence . This process is called the Warburg effect. Tumor cells can grow and progress exponentially through this metabolic process.

In breast tumor cells, there is a significant increase in glycolytic activity. This process involves the overexpression of glycolytic enzymes, including hexokinase 2 (HK2), pyruvate kinase M2 (PKM2), phosphofructokinase (PFK), as well as glucose transporters such as GLUT1 and GLUT2. Conversely, the tricarboxylic acid (TCA) cycle experiences significant dormancy and decreased pyruvate dehydrogenase (PDH) expression. Additionally, the pentose phosphate pathway (PPP) is significantly activated by the hyperactivity of glucose-6-phosphate dehydrogenase (G6PDH) and transketolase (TK). Breast tumors exhibit elevated serine production due to up-regulation of the 3-phospho-glycerate dehydrogenase (PHGDH) enzyme and increased glutamine uptake. The uptake of lipids and cholesterol, along with increased expression of Fatty Acid Synthase (FAS), is also shown in breast cancer cells to facilitate fatty acid production. This metabolic reprogramming is associated with the progression and growth of breast cancer cells.

These alterations include histone modifications such as demethylation of H3K27me3, acetylation of H3k4 in both early and late stages of breast tumors, and acetylation of H4K16, specifically in the early stage. Additionally, elevated levels of H3Kac are associated with overexpression of DNMT1 and Suv4-20. DNA methylation contributes to the silencing of specific genes, while miRNAs and lncRNAs also play various roles in breast cancer from development to progression.

**Table 1:** Effects of different types of ketone bodies on various models of breast cancer

Type of ketone body	Breast cancer model	Dose of ketone body	Observed effect	Ref.
β-Hydroxybutyrate	MDA-MB 231 and MCF-7 cell lines	25 nM	Decreased proliferation	(114)
β-Hydroxybutyrate	MDA-MB231 cells	10 nM	Increased progression.	(115)
β-Hydroxybutyrate	Co-culturing of MCF-7 cell lines and Immortal fibroblast hTERT	10 mM	Up-regulation of enzymes related to Ketone body production and consumption. Increased proliferation of MCF-7 cell	(116)
β-Hydroxybutyrate	Co-culturing of MDA-MB 231 cell lines and Immortal fibroblast hTERT, MDA-MB 231 xenografted mouse model	10 mM for the MDA-MB 231 cell line, 500 mg/kg for the mouse model	Increased proliferation and progression	(117)
β-Hydroxybutyrate	BT20, BT474, HBL100, MCF-7, MDA-MB231, MDA-MB468, T47D cell lines	3 mM	Increased proliferation	(118)
β-Hydroxybutyrate	MCF7, T47D cell lines	10-25 mM	Decreased progression	(119)
Acetoacetate, β-Hydroxybutyrate	MCF-7 cell lines	10 mM	Decreased proliferation	(120)
β-Hydroxybutyrate	Co-culturing and co-injecting MDA-MB-231, MDA-MB-468, MCF-7, MDA-MB-157, MDA-MB-361, and SK-BR-3 cell lines in a mouse model	10-20 mM	Induction of monocarboxylate transporter 2 leading to increased uptake of ketone bodies. Increased progression	(121)
Ketogenic diet	Xenograft mouse model of MDA-MB-468 breast cancer	Protein (8.60%), Fat (75.10%), Fiber (4.80%), Ash (3.00%), Carbohydrate (3.20%)	Increased efficacy of PI3K inhibitors	(123)
Ketogenic diet	Injection of MDA-MB 231 cell lines in Mouse model	10 mM	Increased efficacy of anti-CTLA-4 immunotherapy	(124)
Secretion of ketone bodies by shBRCA1 deficient fibroblasts	Co-culturing MDA-MB-231 cell line and shBRCA1- fibroblasts, Co-injection of MDA-MB-231 cell line and shBRCA1- fibroblasts into a mouse model	Secretion of 0.004 nM ketones by shBRCA1 deficient fibroblasts	Increased mitochondrial activity in tumor cells, Increased proliferation	(125)
Ketogenic diet	Locally advanced and metastatic breast cancer	The patients were given an MCT-based Ketogenic diet (6% carbohydrates, 19% protein, 20% MCT, 55% fat) for 90 consecutive days concurrently with the first three months of chemotherapy	Increased survival rate of patients	(126)
Ketogenic diet	Non-metastasized breast cancer	20 g medium-chain triglycerides per 100 ml + essential amino acids in the form of the MAP supplement, intervention group 2 should follow a whole food KD supplemented with MAP	The body composition, metabolic parameters, and quality of life improved	(127)
Ketogenic diet	Metastatic breast cancer	6% carbohydrate, 19% protein, 20% MCT oil, and 55% fat	Physical activity and the quality of life improved after 6 weeks	(128)
Ketogenic diet	Stage IV metastatic breast cancer	20–50 g/day of carbohydrate, 1.2–1.5 g of protein/kg of reference weight, and dietary fat consumed to satiety	The Ketogenic diet improved metabolic health outcomes	(129)
Ketogenic diet	Stage IV Triple-Negative Breast Cancer	Normal consumption of eggs, leafy greens, above ground vegetables, high fat dairy, natural fats, meats, nuts, and seeds	Combination therapy of a ketogenic diet and chemotherapy improved the quality of life of patients	(130)
Ketogenic diet	Metastatic breast cancer	6% carbohydrate, 19% protein, 20% MCT, and 55% fat	A ketogenic diet decreased tumor size and stage after 12 weeks	(131)

PI3K: Phosphatidylinositol-3 kinase; CTLA-4: Cytotoxic T-lymphocyte-associated protein 4; MCT: Medium chain triglycerides; MAP: Master amino acid pattern; KD: Ketogenic diet

Treatment of various breast tumor cell lines with BHB leads to an increase in H3K9Ac levels. In MCF-7 cells, BHB increases histone acetylation. BHB administration does not affect histone acetylation in a mouse breast tumor model. In MDA-MB-231 cells, BHB treatment increases histone acetylation. Short-term BHB treatment in MDA-MB-231 cells results in histone acetylation and minimal histone butyrylation, while long-term treatment shows no change in histone acetylation or butyrylation.

## Conclusion

The exploration of epigenetic profiles is emerging as a way to understand the molecular mechanisms of breast cancer and to provide strategies for diagnosis, prognosis, and therapy for the disease (81). As described earlier, BHB exerts distinct effects on epigenetic factors in breast cancer cell lines (117, 141-144). Similarly, in animal models of breast cancer, BHB did not affect histone acetylation (142). These findings suggest that BHB’s impact varies significantly depending on the tumor type. Considering the metabolic dependence of cancer cells on glucose, substituting glucose with ketogenic diets offers a promising strategy to hamper tumor growth. However, some studies indicate adverse effects of ketone bodies on cancer cells by promoting invasion and growth. The reason for these contradictory effects of ketone bodies is unknown, but it may be related to epigenetic alterations and the type of tumor. 

It is essential to indicate the significant and sometimes contradictory effects of ketogenic diets on breast cancer. The heterogeneity among breast cancer subtypes necessitates a meticulous consideration of this diversity when evaluating the effect of ketogenic interventions. Researchers should assess various subtypes of this cancer, considering that responses may differ based on the specific characteristics of each subtype. A comprehensive understanding of the intricate interplay between ketone bodies and epigenetic modifications in different subtypes of breast cancer will be critical in seeking therapeutic modalities for the disease. This can help future research to discover personalized and effective interventions for the treatment of breast cancer. This review sheds light on the fact that most studies investigating the impact of ketone bodies on epigenetic alterations in breast tumors have predominantly focused on histone modifications. Besides, we found only six studies investigating the possible effects of ketogenic regimens on breast cancer patients. To the best of our knowledge, no previous study has evaluated the modulating effect of acetone on epigenetic modifications in breast cancer. Only one *in vitro *study evaluated the effect of acetoacetate on a breast cancer cell line (120). Also, the effect of BHB has been studied only on histone modifications in breast cancer. This highlights a critical gap in this research area and necessitates the need for further exploration into some other epigenetic modifications after using ketogenic diets in different models of breast cancer.

## Declaration

We have not used any AI tools or technologies to prepare this manuscript.
